# Causes rares de péritonites sus-mésocoliques: à propos d’une série de 6 cas

**DOI:** 10.11604/pamj.2025.52.156.43640

**Published:** 2025-12-12

**Authors:** Ghita El Baroudi, Mohamed Boulatar, Abdelouahed Louzi, Khalid Rabbani

**Affiliations:** 1 Service de Chirurgie Générale, Hôpital Arrazi, Centre Hospitalo-Universitaire Mohammed VI, Marrakech, Maroc,; 2 Laboratoire de Recherche Morpho-sciences, Faculté de Médecine et Pharmacie de Marrakech, Université Cadi Ayyad, Marrakech, Maroc

**Keywords:** Péritonite sus-mésocolique, abcès hépatique rompu, vésicule biliaire perforée, Supramesocolic peritonitis, ruptured liver abscess, perforated gallbladder

## Abstract

Les péritonites correspondent à une inflammation aiguë du péritoine. Les perforations, ruptures ou nécroses du tube digestif sont responsables de péritonites bactériennes secondaires. Leur traitement comporte une réanimation volémique et respiratoire, une antibiothérapie et une intervention chirurgicale. Les objectifs du traitement chirurgical sont le contrôle du processus pathologique source de la contamination, la réduction de la contamination bactérienne du péritoine, et la prévention de l'infection résiduelle. Nous avons réalisé une étude rétrospective au Service de Chirurgie Générale du Centre Hospitalier Universitaire Mohammed VI de Marrakech, portant sur 6 cas (n = 6) de cause rare de péritonites sus-mésocoliques dont le but est de montrer la difficulté du diagnostic et l'intérêt d'une prise en charge rapide et multidisciplinaire dans le pronostic des péritonites.

## Abstract (en)

Peritonitis is an acute inflammation of the peritoneum. Perforations, ruptures, or necrosis of the digestive tract are responsible for secondary bacterial peritonitis. Treatment includes fluid and respiratory resuscitation, antibiotic therapy, and surgery. The objectives of surgical treatment are to control the pathological process responsible for contamination, reduce bacterial contamination of the peritoneum, and prevent residual infection. We conducted a retrospective study in the General Surgery Department of the Mohammed VI University Hospital in Marrakech, involving 6 cases (n=6) of rare causes of supramesocolic peritonitis, to demonstrate the diagnostic challenges and the importance of rapid and multidisciplinary management in the prognosis of peritonitis.

## Introduction

Les péritonites correspondent à une inflammation aiguë du péritoine dont les causes les plus fréquentes sont infectieuses. Ces affections sont une urgence thérapeutique et nécessitent pour la plupart une prise en charge multidisciplinaire soigneuse, faisant intervenir au minimum le chirurgien, le radiologue, l'anesthésiste-réanimateur et le microbiologiste [1,2]. Ces infections étaient entachées d'une mortalité presque systématique. Les progrès réalisés par les techniques chirurgicales, la découverte des méthodes d'asepsie puis les antibiotiques ont amélioré d'une manière spectaculaire leur pronostic [2].

## Méthodes

**Conception et mise en place de l'étude:** il s'agit d'une étude rétrospective étalée sur une période de quatre ans, de mars 2021 au mois de mars 2024, menée au Service de Chirurgie Générale du Centre Hospitalier Universitaire Mohammed VI de Marrakech ayant inclus toutes les formes de péritonites sus-mésocoliques secondaires à une rupture spontanée de vésicule biliaire et d'abcès hépatique.

**Population étudiée:** un total de 6 cas de péritonite sus-mésocolique dont 4 par perforation spontanée de la vésicule biliaire et 2 par rupture spontanée d'abcès hépatique. Pour toutes nos patientes, le diagnostic de péritonite a été affirmé par la clinique, la biologie et la radiologie et confirmé par la chirurgie. Toutes les autres péritonites sus-mésocoliques par perforation gastrique et post-traumatiques ont été exclues.

**Collecte de données:** une fiche de référence a été utilisée pour collecter les données auprès des dossiers archivés pendant une période de 4 ans au sein du service de chirurgie générale du Centre hospitalier universitaire Mohammed VI de Marrakech. Nous avons précisé pour chaque cas les caractéristiques épidémiologiques, cliniques, biologiques et radiologiques. Nous avons également colligé le résultat et la méthode d'exploration de la cavité abdominale ainsi que les aspects évolutifs après traitement.

## Résultats

La tranche d'âge de nos patients variait entre 50 et 90 ans avec une moyenne de 65,6 ans. On a noté une égalité d'atteinte entre les deux sexes 3F/3H. Tous les patients étaient de bas niveau socio-économique. Aucun n'avait d'antécédents de chirurgie biliaire ou hépatique.

Le signe évocateur chez nos patients était la douleur abdominale d'installation brutale (100%), les vomissements étaient présents chez 50% des cas (n = 3/6), un iléus réflexe chez 33,3% de cas (n = 2/6) et la fièvre chez 4 patients, soit 66,6% (Tableau 1). Deux patients présentaient des signes de choc, soit 33,3%, une contracture abdominale a été objectivée chez 50% de nos cas et 66,6% avaient une distension abdominale associée (Tableau 2). Un syndrome infectieux biologique était présent chez tous nos patients, soit 100%. La tomodensitométrie (TDM) abdomino-pelvienne a confirmé la présence d'un épanchement péritonéal chez tous les patients, la présence d'abcès hépatique chez 2 patients et une cholécystite chez 4 patients (Tableau 3).

**Tableau 1:** manifestations cliniques de péritonites sus-mésocoliques

Signes fonctionnelles	Nombre
Douleur abdominale	6
Vomissement	3
Iléus reflex	2
Fièvre	4

**Tableau 2:** les signes physiques de péritonites sus-mésocoliques

Signes physiques	Nombre
Signes de choc	2
Contracture abdominale	3
Distension abdominale	4

**Tableau 3:** les signes radiologiques de péritonites sus-mésocoliques

Signes radiologiques	Nombre
Epanchement abdominale	6
Abcès hépatique	2
Cholécystite	4

L'exploration chirurgicale a permis de confirmer le diagnostic et a objectivé une perforation spontanée de la vésicule biliaire chez 66,6% (n = 4/6) et une rupture spontanée d'abcès hépatique. Un traitement étiologique avec un lavage et drainage associé à une antibiothérapie a été réalisé chez tous les patients, dont quatre cholécystectomies, soit 66,6% et deux mises à plat d'abcès, soit 33,3%. L'évolution thérapeutique était favorable avec retour à domicile chez 66,6% (n = 4) et décès chez 33,3% (n = 2).

## Discussion

La péritonite aiguë se définit comme une inflammation brutale et diffuse de la séreuse péritonéale d'origine bactérienne ou chimique [3]. Du fait de leur grande hétérogénéité, les péritonites ont suscité plusieurs systèmes de classification:

**Classification de Hambourg** [2,4]**:** c'est la classification la plus utilisée, elle permet de classer les péritonites en: *péritonite primitive* est retrouvée lors d'affections médicales où l'ensemencement de la cavité péritonéale résulte du passage de bactéries par voie hématogène ou par translocation vers la cavité péritonéale. Le traitement de ces péritonites est médical et repose sur l'antibiothérapie. *Péritonite secondaire:* elle est l’une des formes les plus fréquentes de ces affections. Elles sont observées lors d'une perforation du tube digestif ou de l'arbre biliaire, par dissémination des germes digestifs dans le péritoine [1-5]. Le traitement chirurgical est impératif et doit être systématiquement associé à un traitement anti-infectieux. *Péritonite tertiaire:* correspond à une infection intra-abdominale persistante au décours d'une infection initiale documentée. Il s'agit toujours d'une infection secondaire d'évolution compliquée [2,4,6].

**Classification selon la localisation anatomique:** les péritonites peuvent également être différenciées en fonction de leur localisation anatomique. Les infections dues à une lésion à l'étage sus-mésocolique concernent les affections gastriques, duodénales et d'origine biliaire. A l'étage sous-mésocolique, ces infections regroupent toutes les lésions du grêle, appendice, et colon. Cette distinction est basée sur des résultats microbiologiques et des pronostics différents selon ces localisations [1,2].

**Classification selon la sévérité de l'infection:** il est possible de classer les péritonites en fonction de leur sévérité initiale. L'utilisation de scores de sévérité généralistes (score APACHE ou score IGS II) [2,7,8] ou spécialisés (Mannheim Peritonitis Index [MPI] ou le Peritonitis Index Altona [PIA]) [2,9,10] permet de prédire la mortalité de groupes de patients comparables. Cependant, plusieurs travaux ont montré que les patients atteints d'infections sévères avaient un pronostic plus mauvais et nécessitaient une prise en charge plus active en milieu de réanimation.

**Classification selon l'environnement:** enfin, les péritonites sont différenciées selon leur circonstance de survenue. Chez des sujets indemnes de toute pathologie préalable et non hospitalisés, il s'agit d'une péritonite communautaire, également appelée extrahospitalière. L'infection peut être acquise durant le séjour hospitalier ou au décours et elle est alors associée aux soins, il s'agit d'une péritonite nosocomiale [2]. En résumé, les infections les plus fréquentes sont les péritonites secondaires communautaires sus- ou sous-mésocoliques [1,2]. Dans nos cas, les péritonites sont classées parmi les péritonites secondaires communautaires sus-mésocoliques. Le diagnostic de péritonite pose en général peu de problèmes. Il est basé sur des signes cliniques. Le patient se plaint généralement de douleurs abdominales associées à des troubles du transit (nausées, vomissements, arrêt des matières et des gaz, etc.), le plus souvent dans un contexte fébrile. L'examen clinique met en évidence une douleur à la palpation de l'abdomen, une défense en cas d'examen effectué précocement ou une contracture des muscles de la paroi abdominale et une douleur du cul-de-sac de Douglas lors du toucher rectal. Dans nos cas, le signe clinique majeur est la douleur abdominale associée ou pas à des vomissements, ce qui est similaire à la littérature [2,11]. Chez les sujets âgés, les infections intraabdominales peuvent se présenter avec une sémiologie minimale [2,12,13].

Quelque soit l'âge du patient, un diagnostic retardé ou un traitement différé conduisent rapidement à une aggravation du tableau clinique. Des signes biologiques de souffrance tissulaire (élévation de la créatinine, thrombopénie, hypoxémie, ictère ou acidose lactique) sont alors fréquemment constatés, voire un tableau de choc avec défaillance polyviscérale [2,14,15]. Les examens biologiques sont essentiellement utilisés pour évaluer le retentissement de l'infection. Chez les patients âgés, la fréquence de leucopénie (< 2000/mm^3^) paraît accrue par rapport aux sujets jeunes [2,12]. Les autres examens biologiques ne permettent en général pas de s'orienter vers le diagnostic avant le stade de défaillance viscérale. Le bilan biologique permet d'évaluer les besoins de réanimation et est utilisé comme bilan préopératoire. Enfin, la mesure des concentrations de certains marqueurs de l'inflammation (protéine C-réactive, procalcitonine...) a été proposée comme élément du diagnostic par certains auteurs. Tous nos patients présentaient un syndrome infectieux biologique fait d'une hyperleucocytose et d'une *C-reactive protein (CRP)* élevée.

**Les examens radiologiques:** le cliché d'abdomen sans préparation (ASP), l'échographie abdominale et/ou la tomodensitométrie confirment le diagnostic et orientent le geste opératoire. La tomodensitométrie est généralement réservée aux situations complexes ou en cas de doute diagnostique. Dans les formes sévères, la tomodensitométrie ne doit être envisagée que si l'examen ne retarde pas l'intervention. Un examen échographique ou tomodensitométrique « normal » n'élimine pas pour autant le diagnostic.

Certains auteurs proposent le recours à des examens radiographiques digestifs avec un produit de contraste à la recherche d'une fuite extraluminale. Cependant, les résultats de cette opacification n'ont de valeur que lorsqu'ils identifient l'extravasation du produit de contraste. Dans nos cas, l'échographie et la TDM abdominale étaient suffisantes pour poser le diagnostic positif sans recours à d'autres examens radiologiques. Le traitement principal des péritonites repose sur la chirurgie et impose une évaluation complète des lésions et le contrôle de la source infectieuse [1-3,16]. L'antibiothérapie apporte un bénéfice supplémentaire, particulièrement dans les infections sévères, prises en charge tardivement et/ou chez les patients avec des maladies de fond sévères. Les règles fondamentales de la chirurgie septique sont bien établies: exploration abdominale et identification de la source de l'infection, éradication de la cause de l'infection, réduction de la contamination bactérienne par un lavage péritonéal abondant, prévention de la récidive ou de la persistance de l'infection [1,2].

La prise en charge doit être initiée en préopératoire: le bilan préopératoire doit être rapide de façon à ne pas retarder l'intervention. Cette période préopératoire doit être mise à profit pour objectiver et corriger les principales perturbations humorales et stabiliser les déséquilibres hémodynamiques et respiratoires. Quelles que soient les précautions prises, le patient doit être considéré comme hypo volémique et à risque de régurgitation [14,15]. L'indication chirurgicale est formelle et immédiate dès que le diagnostic est suspecté. Seule la chirurgie permet de faire un bilan étiologique complet de l'infection. Le pronostic est directement lié à la rapidité du diagnostic et du traitement [2,16]. L'indication chirurgicale était posée chez tous nos malades.

L'abord chirurgical peut faire appel, en fonction de la pathologie, du terrain et de l'expérience de l'opérateur à une laparotomie ou à une cœlioscopie. L'abord par laparotomie médiane peut être envisagé d'emblée, en cas de contre-indication à la cœlioscopie, si l'état hémodynamique du malade reste précaire. L'incision doit être large de façon à permettre une exploration complète de la cavité abdominale [2,16]. Une laparotomie médiane était la voie d'abord chirurgicale pour tous nos patients. Les prélèvements bactériologiques sont systématiques, l'exploration de la cavité péritonéale implique un contrôle de toutes les régions déclives et de tous les viscères abdominaux, complété par une toilette péritonéale avec lavage abondant (15 à 20 litres) [2]. Un prélèvement peropératoire a été réalisé chez tous les malades pour une étude cytobactériologique. Lorsqu'un geste sur un viscère est rendu nécessaire, on recommande en général une exérèse d'emblée complète du foyer causal de la péritonite. Une hémostase rigoureuse est nécessaire car une collection sanguine en milieu septique expose au risque d'abcès résiduel. Le drainage reste discuté. Il peut s'agir d'un drainage passif par des lames et/ou des drains placés en déclivité. Dans les péritonites secondaires, il est recommandé de réaliser un drainage systématique des régions déclives (sous-phréniques, gouttières pariéto-coliques, cul-de-sac de Douglas…). Il est inutile de réaliser des prélèvements microbiologiques sur ces drainages ouverts [2,17]. Un drainage a été fait chez tous les patients par drain de Redon CH18 au niveau des zones déclives.

L'antibiothérapie contribue à l'amélioration du pronostic. L'antibiothérapie joue particulièrement son rôle dans les premières heures de traitement, en limitant les bactériémies et en réduisant la fréquence de formation des abcès intra-abdominaux résiduels. Le traitement doit être débuté dès que l'indication opératoire est posée. La sélection de l'antibiothérapie relève d'un choix raisonné, orienté par l'examen direct du liquide péritonéal. Le clinicien doit prendre en compte dans le traitement les entérobactéries et les anaérobies même si ces derniers ne sont pas retrouvés. Il n'y a aucun risque de « négativer » les prélèvements peropératoires par une dose initiale d'antibiotique. Le traitement probabiliste doit être réadapté secondairement en fonction des résultats de l'antibiogramme [18-22]. Une bi-antibiothérapie à base de céphalosporine de 3^e^ génération associée à des métronidazoles a été prescrite pour tous les malades.

Le suivi thérapeutique des patients est basé sur l'évaluation des données cliniques et paracliniques. Le traitement complet d'une lésion abdominale évolutive se traduit théoriquement par un retour à une situation clinique normale (apyrexie, disparition de la leucocytose, réapparition du transit) en quelques jours. L'absence d'amélioration ou une aggravation secondaire peut correspondre à l'évolution d'une complication intra- ou extra-abdominale, mais aussi à une défaillance mono- ou polyviscérale, conséquence de la péritonite. Une complication doit être envisagée devant toute évolution clinique anormale. En cas d'échec du traitement antibiotique, l'analyse de cet échec impose de rechercher en premier lieu un problème chirurgical non résolu. Les autres causes d'échec sont dues à un traitement antibiotique inadapté (spectre insuffisant, posologie insuffisante, émergence d'un ou plusieurs germes résistants, sites infectieux inaccessibles aux traitements). En cas de besoin, une laparotomie exploratrice peut être nécessaire pour établir le diagnostic. Dans tous les cas, une reprise chirurgicale inutile est moins dommageable pour le patient qu'une intervention trop tardive [15,23]. Dans nos cas, quatre malades ont bien évolué tandis que deux malades se sont compliqués.

La mortalité des péritonites extrahospitalières varie entre 0 et 50% selon la cause [24]. À la vue des données de la littérature, le terrain paraît jouer un rôle considérable dans le pronostic: âge avancé, pathologies associées, immunodépression, défaillances d'organe, dénutrition. Le retard à l'intervention chirurgicale, source d'accroissement de l'inoculum bactérien, est un facteur de gravité reconnu [15]. Une antibiothérapie initiale inadaptée est aussi un facteur de gravité [25]. Dans notre série, 2 cas sont décédés, soit 33,33%, ce qui rejoint les résultats de la littérature.

## Conclusion

Malgré les progrès réalisés, les péritonites restent une affection sévère, tout particulièrement chez les patients fragiles ou diagnostiqués tardivement. La collaboration avec les équipes de chirurgie digestive, de radiologie interventionnelle et d'endoscopie facilite la prise en charge.

### Etat des connaissances sur le sujet

- Les péritonites sus-mésocoliques sont une urgence médico-chirurgicale vitale;
- Le retard de diagnostic engendre le pronostic vitale.

### Contribution de notre étude à la connaissance

- À penser aux perforations spontanées des abcès hépatiques et des cholécystites aiguës ou chroniques;
- La rapidité du diagnostic positif et de la prise en charge assure une bonne évolution des malades.
